# Digital mental health literacy -program for the first-year medical students’ wellbeing: a one group quasi-experimental study

**DOI:** 10.1186/s12909-021-02990-4

**Published:** 2021-11-06

**Authors:** Marjo Kurki, Gilbert Sonja, Mishina Kaisa, Lempinen Lotta, Luntamo Terhi, Hinkka-Yli-Salomäki Susanna, Sinokki Atte, Upadhyaya Subina, Wei Yifeng, Sourander Andre

**Affiliations:** 1 Department of Child Psychiatry, University of Turku, Lemminkäisenkatu 3, FI-20014 Turku, Finland; 2 Finland INVEST Research Flagship, University of Turku, FI-20014 Turku, Finland; 3 ITLA Children’s Foundation, Porkkalankatu 24, 00180 Helsinki, Finland; 4 Department of Nursing Science, University of Turku, Joukahaisenkatu 3-5, FI-20014 Turku, Finland; 5 Turku University Hospital, PO Box 52, 20521 Turku, Finland; 6 Department of Psychiatry, Faculty of Medicine and Dentistry, University of Alberta, 1E1 Walter Mackenzie Health Sciences Centre (WMC), 8440 112 St NW, Edmonton, AB T6G 2B7 Canada

**Keywords:** Digital intervention, Mental health, Wellbeing, Mental health literacy, Mindfulness, Preventive intervention, Medical student

## Abstract

**Background:**

Medical students are prone to mental disorders, such as depression and anxiety, and their psychological burden is mainly related to their highly demanding studies. Interventions are needed to improve medical students’ mental health literacy (MHL) and wellbeing. This study assessed the digital Transitions, a MHL program for medical students that covered blended life skills and mindfulness activities.

**Methodology:**

This was a one group, quasi-experimental pretest-posttest study. The study population was 374 first-year students who started attending the medical faculty at the University of Turku, Finland, in 2018-2019. Transitions was provided as an elective course and 220 students chose to attend and 182 agreed to participate in our research. Transitions included two 60-minute lectures, four weeks apart, with online self-learning material in between. The content focused on life and academic skills, stress management, positive mental health, mental health problems and disorders. It included mindfulness audiotapes. Mental health knowledge, stigma and help-seeking questionnaires were used to measure MHL. The Perceived Stress Scale and General Health Questionnaire measured the students’ stress and health, respectively. A single group design, with repeated measurements of analysis of variance, was used to analyze the differences in the mean outcome scores for the 158 students who completed all three stages: the pre-test (before the first lecture), the post-test (after the second lecture) and the two-month follow-up evaluation.

**Results:**

The students’ mean scores for mental health knowledge improved (-1.6, 95% Cl -1.9 to -1.3, *P*<.001) and their emotional symptoms were alleviated immediately after the program (0.5, 95% Cl 0.0 to 1.1, *P*=.040). The changes were maintained at the two-month follow up (-1.7, 95% Cl -2.0 to -1.4, *P*<.001 and 1.0, 95% Cl 0.2 to 1.8, *P*=.019, respectively). The students’ stress levels reduced (*P*=.022) and their attitudes towards help-seeking improved after the program (*P*<.001), but these changes were not maintained at the two-month follow up. The stigma of mental illness did not change during the study (*P*=.13).

**Conclusions:**

The digital Transitions program was easily integrated into the university curriculum and it improved the students’ mental health literacy and wellbeing. The program may respond to the increasing global need for universal digital services, especially during the lockdowns due to the COVID-19 pandemic.

**Trial registration:**

The trial was registered at the ISRCTN registry (26 May 2021), registration number 10.1186/ISRCTN10565335).

**Supplementary Information:**

The online version contains supplementary material available at 10.1186/s12909-021-02990-4.

## Introduction

Medical schools worldwide have raised concerns about the mental health of their students, as they face burdens and duties related not only to their demanding curricula and the competitive climate in medical schools and their future profession [1, 2]. Research has shown that medical students have a higher incidence of stress and stress-related mental health problems than the general student population [3–6]. These include self-reported depression and anxiety, reduced sleep quality and burnout, and even suicidal ideation. Medical students also face an elevated risk of non-medical use of prescription medication and illegal drugs, due to the considerable stress [7, 8].

At the same time, medical students encounter notable barriers to seeking help, including lack of knowledge and negative attitudes towards mental disorders and treatment, the fear of being stigmatized and poor access to appropriate care [9, 10]. They may fear that disclosing their mental health problems might jeopardize their professional advancement or cost them their professional rights, and consequently postpone help-seeking [11]. These barriers make it difficult for clinicians to identify problems early enough and provide appropriate treatment. Therefore, strategies to promote mental health and wellbeing need to be incorporated into the medical students’ curricula [12, 13].

Mental health literacy (MHL) is an integral element of health literacy and it contributes to the public health strategies to prevent mental illness and promote mental health [14]. MHL is based on four components: understanding how to obtain and maintain good mental health, understanding mental disorders and their treatment, decreasing stigma and enhancing help-seeking behavior [15, 16]. MHL increases mental health knowledge and positive attitudes towards the services available for mental health issues and lowers the threshold for seeking help and using effective treatment [17]. In contrast, poor MHL may lead to increased stigma, lack of awareness of how to identify mental disorders, and barriers to seeking help, namely confidentiality and trust in potential source of help. These situations have been associated with compromised wellbeing, quality of life and performance [18].

Previous studies have shown that MHL programs for adolescents in high-school settings can increased mental health knowledge and skills [19, 20]. But a systematic review reported that university-based mental health educational programs did not improve attitudes towards seeking help or stigma among students studying to be health professionals [21].

From a developmental perspective, moving from the family environment to independent living is a critical transition period in a young adults’ life, especially when this is coupled with the academic pressures of university studies. Strategies that are integrated into university settings, such as MHL interventions, can increase mental health awareness and reduce the stigma associated with mental health problems. This promotes wellbeing [13, 22]. In the UK, an evidence-based Mental Health First Aid e-learning course, which focused on general awareness of mental health and recognizing mental health problems and mental disorders, was provided for medical students. This demonstrated promising results in terms of improved MHL [23]. The course also improved the participants’ attitudes to providing help to those with mental health conditions [21]. The Canadian Transitions program, which provided the basis for the Finnish model, combines MHL with comprehensive life-skills resource for young people when they are making the transition to university studies [24]. In 2021, Wei et al. (2021) compared the findings of first-year postsecondary students who participated in the Canadian Transitions intervention and a control group who did not two months after the program ended [25]. The students in the intervention group showed improved mental health knowledge, reduced stigma, improved positive attitudes towards help-seeking, increased help-seeking behavior and reduced stress. Moreover, the participants felt more prepared for their academic studies after the program [26].

Other approaches have been used for medical students in addition to MHL programs. For example, mindfulness exercises effectively reduced stress, anxiety, depression and mental suffering among medical students, by increasing awareness, skills, efficiency and well-being [27].

This study was adapted from the Canadian Transitions initiative and included extra mindfulness exercises for stress management [24, 25]. Our participants were first-year medical students who had enrolled at the University of Turku, Finland, at the start of the 2018 and 2019 academic years and the aim was to promote their well-being. Our hypothesis was that the digital Transitions program would improve their knowledge about mental health, decrease the stigma associated with mental health problems, improve help-seeking attitudes and reduce their perceived stress and emotional symptoms.

## Methods

### Study design and participants

**Fig. 1:**
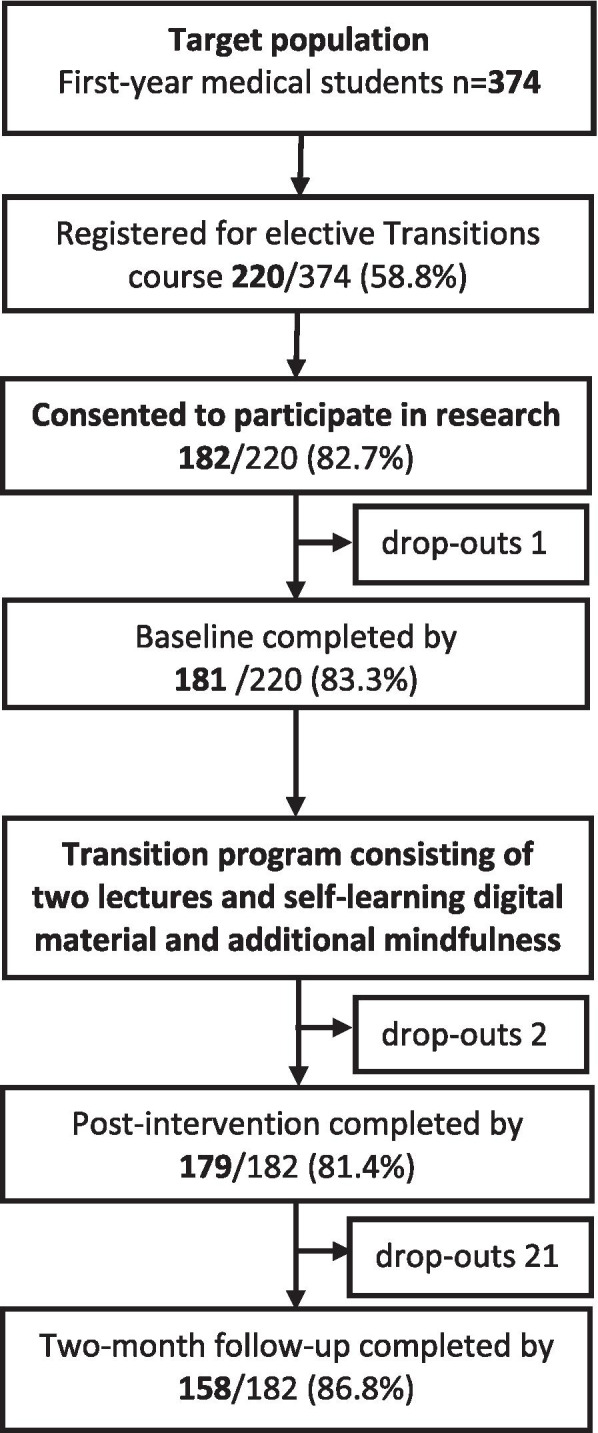
Flow chart for the study

The study was conducted using a one group, quasi-experimental pretest-posttest design. A universal digitalized Transitions program was integrated into the first-year studies of the medical faculty at the University of Turku, South-Western Finland, as an elective course. The study population comprised 374 general medicine and dentistry students who started their studies in the 2018-2019 academic years. The 220 students who selected the course registered on the Transitions Internet-based platform. The study sample consisted of the 182 students who agreed to participate in our study, as shown in the flow chart (Fig. 1). All the participants received the intervention and there was no control group, because only students from one study site were available. Participation in the study was voluntary and the students could also complete the course without participating in the research.

The inclusion criteria for the participants were that they were first-year medical or dentistry students at the University of Turku in the 2018 and 2019 academic years, that they selected Transitions as an optional course and self-registered on the program website and they provided informed consent to participate in the research. The study was approved by the Ethics Board of human sciences research at the University of Turku, Finland.

### Program content

The Canadian Transitions program was originally a booklet, and it was published online on 21 May 2019 at https://mentalhealthliteracy.org/product/transitions/. The program material was translated into Finnish by a professional translator and culturally adapted and digitalized by staff at the Research Center for Child Psychiatry at the University of Turku, Finland. Multi-professional experts reviewed the adapted material. These included adolescent psychiatrists, specialists in sexual diseases, substance abuse and communication difficulties, a teacher who specialized for learning strategies and 10 university students. Their feedback was carefully considered. Cultural adaptation and digitalization shortened the original material. Some of it was provided as videos, whereby students and professionals provided tips and advice for studying. The program also provided links to further information on specific topics, such as webpages run by Finnish mental health organizations.

The contents of the digital Transitions material focused on three themes that addressed life skill resources and mental health topics (Table 1). Theme one focused on important skills for independent living, academic life strategies and relationships. Theme two provided strategies for how to obtain and maintain sound mental health and stress management skills. Theme three concentration on mental disorders, related treatment and help seeking. The material was presented as educational text and tips, with videos based on students’ experiences and links to useful information sources. Multiple choice questions related to the topics were presented after each theme. Students needed to answer these questions to move to the next theme and complete the post-intervention questionnaires.

**Table 1:** Contents of the digital transitions program

The first 60-minute lecture introduced one and two. It encouraged the participant to study the digital material and use the stress management techniques.		
Independent learning of the digital Transitions material for 4 weeks		
**Theme 1**		
**Independent life** My finances Moving to my own apartment Living alone or with others? Family support	**Study skills** Time management Optional studies Preparing for exams Managing exam stress Self-esteem affects learning Learning difficulties	**Relationships** Loneliness and new relationships End of dating Sexuality Harmful relationships Bullying and sexual harassment Sexual violence Drug-facilitated sexual assault Anger and violence Violence in relationship
**Theme 2**		
**Mental health is a resource** What is mental health? Improve your mental wellbeing	**Stress** Stress management techniques Crises	**Intoxicants** Responsible alcohol use Information on drugs Stop gambling
**Theme 3**		
**Mental disorders** Depression Anxiety disorders Eating disorders Substance abuse disorders Self-harm Bipolar disorders Psychoses	**Help-seeking** When to seek help? Where to get help? Mental health or addiction problems affecting love one	**Treatment** Talking with a professional Medication Psychotherapy Other treatments Supporting the loved ones
The second 60-minute lecture introduced Theme three. It aimed to enhance the participant’s self-learning and promote help-seeking behavior.		

The mindfulness component was an additional stress management resource. It included a series of audio tapes: 10 sessions that lasted from 4-10 minutes covered the theory of mindfulness and instructions for how to apply it and 10 sessions that lasted from 5-30 minutes that focused on exercises. The exercises were based on mindfulness-based stress reduction (MBSR) and mindfulness-based cognitive therapy (MBCT) –programs, but were specifically modified for this age group by a mindfulness instructor at the University of Turku.

### Program procedure

The courses started approximately after one month of the first semesters in 2018 and 2019. The students who selected the course registered on the Transitions program website using the link that was emailed to them. If they wanted to participate in the study, they provided their informed consent and completed the electronic baseline questionnaires. The electronic questionnaires at all stages of the research were filled by the participants.

Two 60-minute face-to-face lectures were delivered by a mental health professional. The first lecture marked the beginning of the program, and it focused on strategies for independent living and studying (Table 1). The students then had approximately four weeks to independently learn the material on the digital platform. The course corresponded to one European Credit Transfer System credit, which is the equivalent of a student working 27 hours. The students were required to allocate their time for the independent learning as part of the program. The students were also encouraged to practice stress management skills, including the mindfulness exercises on the platform, at any time during the course. The second lecture was held at the end of the program and was focused on mental health and stress management, as well as mental disorders, help-seeking and treatment. The post-intervention evaluation was conducted immediately after the second lecture (Table 1). The students received an automatic e-mail from the platform two months after the program started to notify them that the follow-up questionnaires were open. They were contacted by e-mail and/or telephone to remind about the questionnaires.

## Measurements

### Background variables

The students provided their name, e-mail address and phone number during the registration process. The baseline evaluation included questions about the following demographic characteristics: Discipline, birth year, gender, whether and when they had moved from elsewhere to Turku to pursue their studies, their current type of accommodation and whether they had sought help for mental health problems in the last three months. They were also asked about which of the program topics they needed to know about, such as study skills, finances, life management, accommodation, mental health, relationships, mental health problems and substance abuse.

### Outcomes

The outcomes came from six questionnaires, which were identical in the baseline, post-intervention and follow-up evaluations. Mental health literacy was measured using three primary outcomes which addressed three separate dimensions: knowledge about mental health, stigma related to mental health problems and help-seeking attitudes [28]. These questionnaires were modified from the mental health literacy questionnaires developed by Kutcher et al. [29–31]. Cultural adaptation and digitalization of the original Transitions material meant that the content of the Finnish program was shorter than the original Canadian program and the questionnaires were modified to correspond to the digital contents.

*The Mental Health Knowledge* questionnaire consisted of 13 statements that addressed the students’ understanding of life skills, mental health, mental health problems and mental disorders. For example, one statement said: a small amount of anxiety could help how well a student performed at a sporting event or on a test [29]. The scores were based on a multiple choice response scale: true, false, don’t know. Each correct response scored one point and each incorrect or ‘don’t know’ response scored zero. The total knowledge score ranged from 0-13. The knowledge questionnaires yielded a Cronbach alpha of .60 for the pooled student data in 2018 and 2019.

The Stigma questionnaire comprised 12 statements, which measured the students’ attitudes towards mental health and mental illness. For example, one statement said: a person who received mental health treatment was just as intelligent as an average person [30]. Each statement was scored using 1-5 on a Likert scale. The total score for each participant ranged from 12-60. Higher scores indicated more positive attitudes and lower stigma. The Stigma questionnaire yielded a Cronbach alpha of .66 for the pooled data.

*The Help-seeking* questionnaire comprised five statements that covered attitudes towards help-seeking for mental health problems. For example, one statement said: asking for help with a mental health problem or disorder was generally helpful [31]. Each statement was assigned a value from 1-5 on a Likert scale [ 24, 26] and the participants received a total score of 5-25, with higher scores representing more positive attitudes towards seeking help. The Help-seeking questionnaire yielded a Cronbach alpha of .67 for the pooled data.

*The General Health Questionnaire* was used to measure health and emotional symptoms, mainly anxiety and depression [32]. It contained 12 statements and each response assigned a value between 0-3 on a Likert scale. Total score ranged from 0-36, with higher scores indicating more severe health concerns. The questionnaire has previously been reported to have strong reliability and validity, with a Cronbach alpha of .88 [25].

*The Perceived Stress Scale* (PSS) was used to measure student’s self-reported stress [33]. The instrument consists of 10 statements and each response scored a value between 0-4 on a Likert scale. Total scores ranged between 0 and 40, with higher scores indicating higher levels of perceived stress. Previous studies have reported strong reliability and validity for the questionnaire [34]. Cronbach alpha of .79 was reported in a recent study [25].

*The Client Satisfaction Questionnaire (CSQ-I)*, an instrument designed for digital health interventions [35], was modified and applied in the post-intervention evaluation. This measured the students’ satisfaction with the Transitions program. Five questions were adapted from the CSQ-I and these included whether the students found the program useful and whether they would recommend it to a friend. Each question scored 1-5 on a Likert scale and this generated a total score of 5-25. A Cronbach alpha of .88 was calculated for the present data. All instruments were translated and back-translated, according to a good scientific practice [36].

### Statistics

All 158 students who filled in the pre-test, post-test, and follow-up questionnaire, were included in the analysis. The mean scores of the primary and secondary variables were analyzed according to a single group design with repeated measurements analysis of variance (RM ANOVA). Interaction effects with the background and outcome variables were tested within the repeated measurement ANOVA models. The only interaction effects that were significant were gender, whether students had moved from outside the area to attend the course and the year of the course (2018 or 2019). This meant that the linear mixed model that analyzed the primary and secondary variables included the time, namely baseline, post-test and follow up, as the within-factor. Meanwhile, gender, year of the course and whether they had moved from elsewhere were used as between-factors. We used an unstructured covariance structure that allowed us to include estimates of co-variances within subjects as well as between subjects. The normal assumption of using the linear mixed modelling approach was checked with residual plots. As the restricted maximum likelihood estimation method was applied, there was no need for imputation. A two-sided significance level of 0.05 was used during the statistical testing and 95% confidence intervals (95% Cl) were calculated for the point estimates. Where appropriate, the Bonferroni-correction was applied to counteract the problem of multiple comparisons. The statistical analyses were carried out with SAS statistical software (SAS 9.4, SAS Institute, Cary, NC, USA).

## Results

More than half of the first-year medical students, 58.8% (220/374), chose the optional course and registered with the program, and 82.7% (182/220) of those participated in the study. The drop-out rate was 13.2% (24/182). The follow-up questionnaire was filled in by 86.8% (158/182) of the participants (Fig. 1). Of the 158 participants (74.0% female), 42.4% (67/158) participated in 2018 and 57.6% (91/158) in 2019. The majority (82.3%) studied general medicine, while 17.7% studied dentistry. Most participants (71.5%) had moved from elsewhere less than a year ago, and currently lived alone (65.8%) (Table 1). About a quarter (24.7%) of the participants had already contacted, or planned to contact, a healthcare professional about mental health problems (Table 2). There were no differences in the background characteristics of the students who completed all evaluations and those who dropped out before the post-intervention or follow-up evaluations (Supporting material, Table 1).

**Table 2:** The baseline background characteristics of the 158 participants who completed all stages of the study

Variable	Count (%) *n*=158
Year	
2018	67 (42.4)
2019	91 (57.6)
Gender	
Female	117 (74.0)
Male	41 (26.0)
Age	
18-21 years	108 (68.3)
22-26 years	50 (31.7)
Discipline	
General medicine	130 (82.3)
Dentistry	28 (17.7)
Moved from another municipality	
Yes	113 (71.5)
No	45 (28.5)
If yes, when?	
Within previous year	94/113 (83.2)
More than a year ago	19/113 (16.8)
Type of accommodation	
Alone	104 (65.8)
With a roommate	11 (7.0)
With a partner	33 (20.9)
With parents	10 (6.3)
Help-seeking for mental health problems in previous three months	
No problems requiring help	109 (69.0)
Considered or actually sought help	39 (24.7)
Would not seek help if had problems	10 (6.33)

The students spent approximately an hour and a half engaging in the Transitions program with a median of 96.0 minutes and interquartile range (IQR) of 403.0–193.2 minutes. More time was spent on theme one (median 52.0 minutes, IQR 8.0–127.0 minutes, missing observations three) than on theme 2 (median 2.4 minutes, IQR 1.4–16.2 minutes, missing observations 16) and theme three (median 4.4 minutes IQR 1.4–30.8 minutes, missing observations 20).

**Fig. 2:**
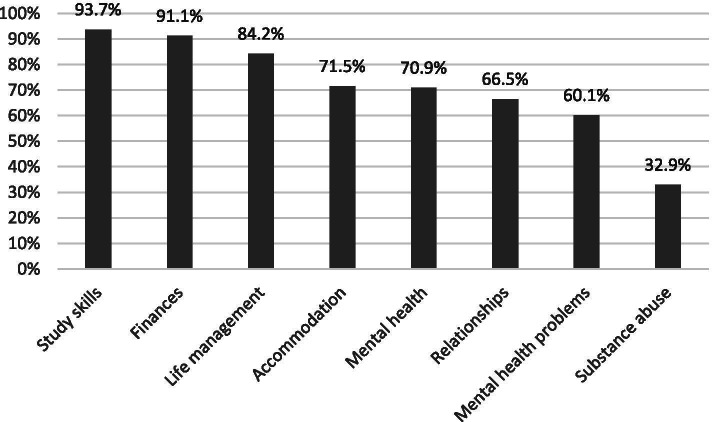
The self-reported baseline needs of 158 first-year medical student in relation to the knowledge they wanted on the different topics covered by the Transitions program

At baseline, most students felt that they needed greater knowledge to be able to handle their studies and life skills, their finances, their accommodation and relationships, as well as mental health and mental health problems. Only about a third of the students needed information about substance abuse (Fig. 2).

Table 3 shows the mean values at baseline, after the intervention, and at follow up. Table 4 shows the changes between these time-points, with regards to knowledge, stigma, attitudes towards help-seeking, perceived stress, and emotional symptom scores. The students’ knowledge about mental health and their emotional wellbeing, improved significantly immediately after the program (*P*<.001 and *P=*.04, respectively), and those positive changes were maintained at the follow-up stage. Furthermore, the students’ attitudes towards help-seeking improved, and they reported reduced stress levels immediately after the program (*P*<.001 and *P*=.022, respectively). However, these changes were not maintained at follow up. There were no changes in stigma.

**Table 3:** Averages scores of the 158 participants with regard to mental health knowledge, stigma, help-seeking attitudes, perceived stress and emotional symptoms at baseline, post-test and follow up

Variable	Baseline Mean^a^ (SE)	Post-test Mean^a^ (SE)	Follow-up Mean^a^ (SE)
Knowledge	7.5 (0.2)	9.1 (0.2)	9.2 (0.2)
Stigma	54.2 (0.3)	54.1 (0.5)	53.6 (0.5)
Help-seeking attitudes	21.6 (0.2)	22.1 (0.2)	21.9 (0.3)
Perceived stress	10.6 (0.6)	9.8 (0.6)	9.9 (0.6)
Emotional symptoms	11.1 (0.6)	10.5 (0.5)	10.1 (0.5)

^a^Model based least squares means. Adjusted for year, gender, moved from elsewhere, and year when they started their studies and help-seeking. *SE* standard error

**Table 4:** Average changes reported by the 158 participants, from baseline to post-test and follow up, with regard to scores on knowledge, stigma, help-seeking attitudes, perceived stress and emotional symptoms

Variable	Baseline to post-test		Post-test to follow up	Baseline to Follow up		
	Mean^1^ (95% CI)	***P*** value^2^	Mean^1^ (95% CI)	***P*** value^2^	Mean^1^ (95% CI)	***P*** value^2^
Knowledge	-1.6 (-1.9 to -1.3)	<.001	-0.1 (-0.3 to 0.2)	.65	-1.7 (-2.0 to -1.4)	<.001
Stigma	0.1 (-0.7 to 0.8)	>.90	0.5 (-0.4 to 1.5)	.28	0.6 (-0.2 to 1.3)	.13
Help-seeking attitudes	-0.5 (-0.8 to -0.2)	<.001	0.2 (-0.2 to 0.5)	.39	-0.3 (-0.7 to 0.0)	.07
Perceived stress	0.8 (0.1 to 1.4)	.022	-0.1 (-0.9 to 0.6)	.77	0.7 (-0.1 to 1.4)	.09
Emotional symptoms	0.5 (0.0 to 1.1)	.040	0.5 (-0.3 to 1.3)	.26	1.0 (0.2 to 1.8)	.019

^1^Model based least squares means. Adjusted for year, gender, moved from elsewhere and seeking help

^2^Bonferroni-adjusted *P* values

**Fig. 3:**
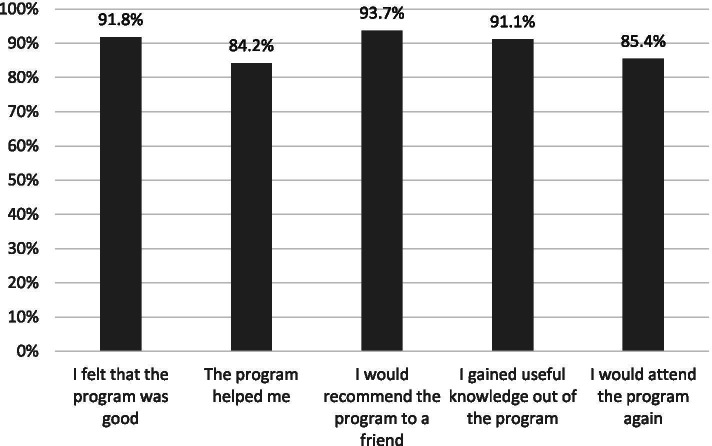
Satisfaction in the various aspects of the digital Transitions program reported by the 158 participants

Out of the 158 participants, 91.8% were satisfied with the digital Transitions program and the vast majority found the program useful and helpful for them. They were also willing to attend it again and would recommend it to a friend (Fig. 3).

## Discussion

To our knowledge, this was the first study to combine a digitally delivered MHL program with a mindfulness component for medical students. We found that after they had participated in the digital Transitions program, the students’ knowledge about mental health increased, their and emotional symptoms were alleviated, and these improvements were maintained for two months. Furthermore, their help-seeking attitudes improved, but only in a short-term, as the increase from baseline to the follow up stage was only close to statistically significant. The students also reported less stress immediately after the program. No changes in stigma were found. Students were very satisfied with the Transitions program.

Our finding that the MHL of the medical students improved after the Transitions program agreed with previous studies, which suggested that mental health knowledge can be improved by delivering MHL or mental health first aid programs in school and university settings [19–21, 37, 38]. Evidence from controlled trials also shows that MHL courses decreased stigma and improved attitudes towards help-seeking in a various settings, including medical schools [19–21]. The effect could be sustained for up to six months according to a systematic review [38]. However, the results about decreased stigma and improved attitudes to help-seeking are controversial. For instance, Wei et al. showed that, in addition to improved mental health knowledge, stigma was reduced and the help-seeking attitudes and behavior of post-graduate students improved [25]. Small reductions in stigma were also observed in a systematic review and meta-analysis of MHFA studies. In addition, participants who attended MHFA courses reported it increased their confidence in helping people with mental health problems and providing mental health first aid. However, a systematic review of studies that focused on mental health educational programs found no significant improvements in help-seeking attitudes or stigma among healthcare students [21]. The original Canadian Transitions program, which was provided as a printed and online booklet, yielded similar positive outcomes to our current study [24, 25]. However, it found that stigma was reduced by the program, and we did not observe any change in this factor. This could be due to a ceiling effect, which refers to a situation where the study subjects score close to the maximum score at baseline, which means there is little room for improvement. That was the case in the present study, which focused on an optional course. We believe that our Transitions program was probably was chosen by students with high mental health awareness to begin with. In general, the stigma surrounding mental health problems and attitudes towards help-seeking vary a lot among countries. Some studies have indicated that, although the stigma related to mental disorders still remains in Finland, its citizens may hold more positive attitudes than those living in other European countries [39].

The students reported significantly lower stress levels and decreased emotional symptoms immediately after the program than before it. Stress is known to adversely affect the medical students worldwide throughout their studies. Levels have been reported to remain moderately high throughout the first three years, with simultaneously worsening of physical, emotional and overall health during the first year [40, 41]. Studies have reported that the prevalence of stress among medical students has ranged between 21 and 90% and that this was often associated with the competitive atmosphere in medical schools [42, 43]. It is important to note that our study found that the students’ stress levels and emotional symptoms improved after the digital Transitions program. Stress management skills are very important for medical professionals, not just during education, but in an individual’s working life, as they can face of major importance not only during the studies but also later in the working life, as they can face daily situation that provoke stressful and emotional responses. Improved stress management skills can lead to better working performance and satisfaction as a medical doctor after medical school [2, 42]. Our findings are an effective response to the international call for evidence-based interventions to improve the mental health of vulnerable groups, such as children and young people, including students [44].

One of the encouraging findings of this study was that the students reported improved emotional wellbeing after the program and this change was also seen two months after the program ended. It is notable that the Canadian study included university students from various faculties and no changes in emotional symptoms after the program were observed [25]. In the present study, the lectures emphasized the importance of empowering the students. They were strongly encouraged, and motivated, to exercise stress management skills. We also encouraged them to identify and reflect on factors that affected their personal wellbeing and what they could do to improve it. The resources that were added to the digital platform may have provided extra help on how to cultivate personal wellbeing.

The original Canadian Transitions program, and our digitalized version of the Finnish Transitions program, were aimed at the first-year university students who were making the transition to independence. This target audience differed from other MHL programs. Both the original Canadian program, and the digitalized Finnish Transitions program, covered a wider rage of more general topics than other studies. These included positive and harmful relationships, loneliness, financial concerns, academic work loads, time management and pressure to perform. All of these have been identified as primary stressors among medical students in qualitative and quantitative assessments [40]. As shown in Fig. 2, the medical students in our study reported that they primarily needed knowledge on study skills, finances, life skills and accommodation, but they also needed information on mental health topics.

Our findings suggest that the digital Transitions program was a feasible method of providing medical students with blended life skill and MHL and the participants were very satisfied with the program. Digital delivery enabled us to provide embedded features, such as links to appropriate further reading, mindfulness audio tapes and videos. The program was easily adopted by the students, due to its clear structure, recurring topics and pragmatic tips, and this advice was easily applied in their daily lives. The key contents and skills, such as the nature of stress and how to manage it, were emphasized were emphasized during the two face-to-face lectures that accompanied the digital program. The relatively short visits to the website did not reflect the entire time invested by the students into learning about mental health. It is likely that the mindfulness exercises, and the other exercises provided by the program, help them to manage their stress and that these contributed to the other positive impacts of the program.

### Strengths and limitations

The main strength of the study was the digital delivery, which enabled us to provide videos and links to enhance learning. Moreover, the Transitions program contained learning material that ranged from general life skills to specific information about mental health. The survey carried out at the start of the program indicated that although the students needed more information about mental health and the problems it could cause. However, they also wanted to know about more general topics, including academic and life skills was highlighted. The holistic design of the program may partly be reflected in the improvements observed in the main outcomes.

The main limitation of the study was that it did not include a control group and this means that we cannot draw solid conclusions about the effectiveness of the intervention. One group pretest and posttest designs have been criticized because they are not suitable for determining causality. Problems have also been reported about internal consistency. However, the study design continues to be applied in various contexts that study the implementation of behavioral interventions, for example in social sciences [45]. The approach in this study was applied because only one group was available. Clustered randomization would have been required to reliably divide the students into treatment and control groups, which was not feasible. We observed a significant change in four outcomes after the program was implemented, and the students’ feedback on the program was very good. The students spent relatively short time on the Transitions program website. This raises questions about whether the significant positive changes in the main outcomes were related to self-learning of the program contents or whether the improvements could reflect the situation that the students had acclimatized to their new life situation as students. Although the main educational method used by the program was self-learning, the key contents of the Transitions program were delivered to the students in two lectures, and these were compulsory for those who opted to take part in the program. This provided the essential knowledge they needed on the topics covered by the program. It was not possible to carry out separate analyses of the impacts of the two compulsory lectures and self-learning on the main outcomes, especially with regards to knowledge about mental health. However, it is likely that some of the knowledge was gained during the lectures and this validates the findings to some degree.

Integrating MHL courses into curricula may be one approach to promoting the wellbeing and mental health of medical students. In this study, the medical students, who selected the digital Transitions as an optional course, were very satisfied with it. Their MHL and emotional wellbeing improved and their stress reduced after the program. We recommend that the digital Transitions program should be provided as a mandatory part of curricula when Finnish medical students start their studies. Transitions program is a universal MHL program and it could be easily adapted to students in other contexts. Future studies should focus on implementing the program outside medical schools, in other faculties and in an internationally. Such studies are particularly topical at the moment, as the need for preventive interventions has increased globally due to the COVID-19 pandemic.

## Conclusion

Digital Transitions was a feasible program for increasing the MHL of first-year medical students in Finland and satisfaction with the intervention was high. We suggest that the program may form a mandatory part of the curricula for medical students and it could be expanded to students in other contexts and countries.

## Supplementary Information

**Additional file 1: Supplementary Table 1.** Background characteristics at baseline of all participants who filled baseline evaluation (*n*=182) and the participants who filled baseline, post-intervention and follow-up evaluations (*n*=152).

## Data Availability

The datasets generated during and/or analyzed during the current study are not publicly available due to the sensitive nature of the personal data of the study subjects. The data are protected by the privacy regulations of European Union *General Data Protection Regulation.*

## Abbreviations

MHL: Mental health literacy
MBSR: Mindfulness-based stress reduction
MBCT: Mindfulness-based cognitive therapy
PSS: Perceived Stress Scale
CSQ-I: Client Satisfaction Questionnaire
RM ANOVA: Repeated measures analysis of variance
IQR: Inter-quartile range

## References

[1] de Almeida GC, de Souza HR, de Almeida PC, de Almeida BC, Almeida GH (2016). The prevalence of burnout syndrome in medical students. Rev Psiquiatr Clin.

[2] Boni RADS, Paiva CE, de Oliveira MA, Lucchetti G, Fregnani JHTG, Paiva BS. Burnout among medical students during the first-years of undergraduate school: prevalence and associated factors. PLoS One. 2018. 10.1371/journal.pone.0191746. 10.1371/journal.pone.0191746 PMC5841647 29513668

[3] Heinen I, Bullinger M, Kocalevent RD. Perceived stress in first year medical students - associations with personal resources and emotional distress. BMC Med Educ. 2017. 10.1186/s12909-016-0841-8. 10.1186/s12909-016-0841-8 PMC5216588 28056972

[4] Ludwig AB, Burton W, Weingarten J, Milan F, Myers DC, Kligler B. Depression and stress amongst undergraduate medical students. BMC Med Educ. 2015. 10.1186/s12909-015-0425-. 10.1186/s12909-015-0425-z PMC4551568 26311538

[5] Wolf MR, Rosenstock JB. Inadequate sleep and exercise associated with burnout and depression among medical students. Acad Psychiatry. 2017. 10.1007/s40596-016-0526-y. 10.1007/s40596-016-0526-y 26976402

[6] Jacob R, Li T-Y, Martin Z, et al. Taking care of our future doctors: a service evaluation of a medical student mental health service. BMC Med Educ. 2020. 10.1186/s12909-020-02075. 10.1186/s12909-020-02075-8 PMC7257172 32471406

[7] Abbasi-Ghahramanloo A, Fotouhi A, Zeraati H, Rahimi-Movaghar A. Prescription drugs, alcohol, and illicit substance use and their correlations among medical sciences students in Iran. Int J High Risk Behav Addict. 2015:e21945. 10.5812/ijhrba.21945 PMC4360541 25821750

[8] Papazisis G, Tsakiridis I, Siafis S. Nonmedical use of prescription drugs among medical students and the relationship with illicit drug, tobacco, and alcohol use. Subst Abus. 2018. 10.1177/1178221818802298. 10.1177/1178221818802298 PMC6149012 30262985

[9] Thompson A, Hunt C, Issakidis C (2004). Why wait? Reasons for delay and prompts to seek help for mental health problems in an Australian clinical sample. Soc Psychiatry Psychiatr Epidemiol.

[10] Vorma H, Rotko T, Larivaara M, Kosloff A (2020). National Mental Health Strategy and Programme for Suicide Prevention; 2020–2030.

[11] Mehta SS, Edwards ML. Suffering in silence: mental health stigma and physician’s licensing fears. Am J Psychiatry Resid J. 2018. 10.1176/appi.ajp-rj.2018.131101.

[12] Seritan A, Hunt J, Shy A, Rea M, Worley L (2012). The state of medical student wellness: a call for culture change. Acad Psychiatry.

[13] Ishak WW, Nikrawesh R, Lederer S, Perry RI (2020). Burnout in medical students. Clin Teach.

[14] Kickbusch I, Pelikan, JM, Apfel F, Tsouros AD. World Health Organization. Health literacy: the solid facts. 2013. http://www.euro.who.int/__data/assets/pdf_file/0008/190655/e96854.pdf. Published 2013. Accessed 26 Jan 2021.

[15] Kutcher S, Wei Y, Coniglio C (2016). Mental health literacy: past, present, and future. Can J Psychiatr.

[16] Kutcher S, Wei Y, Costa S, Gusmão R, Skokauskas N, Sourander A. Enhancing mental health literacy in young people. Eur J Child Adolesc Psychiatry. 2016. 10.1007/s00787-016-0867-9. 10.1007/s00787-016-0867-9 27236662

[17] Rüsch N, Evans-Lacko S, Henderson C, Flach C. Knowledge and attitudes as predictors of intentions to seek help for and disclose a mental illness. Psychiatr Serv. 2011. 10.1176/appi.ps.62.6.675. 10.1176/ps.62.6.pss6206_0675 21632739

[18] Gulliver A, Griffiths KM, Christensen H. Perceived barriers and facilitators to mental health help-seeking in young people: a systematic review. BMC Psychiatry. 2010; http://www.biomedcentral.com/1471-244X/10/11. 10.1186/1471-244X-10-113 PMC3022639 21192795

[19] Milin R, Kutcher S, Lewis S, Walker S, Wei Y, Ferrill N, Armstrong M (2016). 2016. Impact of a mental health curriculum on knowledge and stigma among high school students: a randomized controlled trial. J Am Acad Child Adolesc Psychiatry.

[20] Tehrani H, Olyani S (2021). The effect of an education intervention on mental health literacy among middle school female students. J Health Literacy.

[21] Lo K, Gupta T, Keating JL (2018). Interventions to promote mental health literacy in university students and their clinical educators. a systematic review of randomized control trials. Health Prof Educ.

[22] Mcluckie A, Kutcher S, Wei Y, Weaver C. Sustained improvements in students’ mental health literacy with use of a mental health curriculum in Canadian schools. BMC Psychiatry. 2014. 10.1186/s12888-014-0379-4. 10.1186/s12888-014-0379-4 PMC4300054 25551789

[23] Davies BE, Beever E, Glazebrook C. A pilot randomised controlled study of the mental health first aid eLearning course with UK medical students. BMC Med Educ. 2018. 10.1186/s12909-018-1154-x. 10.1186/s12909-018-1154-x PMC5863362 29562906

[24] Potvin-Boucher J, Szumilas M, Sheikh T, Kutcher S (2010). Transitions: A mental health literacy program for postsecondary students. J Coll Stud Dev.

[25] Wei Y, Kutcher S, Austen E, et al. The impact of transitions, a mental health literacy intervention with embedded life skills for postsecondary students: preliminary findings from a naturalistic cohort study: L’effet des transitions, une intervention de littératie en santé mentale avec compétences essentielles intégrées pour des étudiants du post-secondaire : résultats préliminaires d’une étude de cohorte naturaliste. Can J Psychiatry. 2021. 10.1177/07067437211037131. 10.1177/07067437211037131 PMC9152239 34379024

[26] Kutcher S, Wei Y, Morgan C. Mental health literacy in post-secondary students. Health Educ J. 2015. 10.1177/0017896915610144.

[27] Daya Z, Hearn J. Mindfulness interventions in medical education: a systematic review of their impact on medical student stress, depression, fatigue and burnout. Med Tech. 2018. 10.1080/0142159X.2017.1394999. 10.1080/0142159X.2017.1394999 29113526

[28] Wei Y, McGrath P, Hayden J, Kutcher S (2015). Mental health literacy measures evaluating knowledge, attitudes and help-seeking: a scoping review. BMC Psychiatry.

[29] Wei Y, McGrath P, Hayden J, Kutcher S. Measurement properties of tools measuring mental health knowledge: a systematic review. BMC Psychiatry. 2016. 10.1186/s12888-016-1012-5. 10.1186/s12888-016-1012-5 PMC4995619 27553955

[30] Wei Y, McGrath P, Hayden J, Kutcher S. The quality of measurement tools evaluating the stigma of mental illness: a systematic review. Epidemiol Psychiatr Sci. 2017. 10.1017/S2045796017000178. 10.1017/S2045796017000178 PMC6999021 28462747

[31] Wei Y, McGrath P, Hayden J, Kutcher S. Measurement properties of mental health literacy tools measuring help-seeking: a systematic review. J Ment Health. 2017. 10.1080/09638237.2016.1276532. 10.1080/09638237.2016.1276532 28355928

[32] Goldberg (1978). Manual of the general health questionnaire.

[33] Cohen S, Kamarck T, Mermelstein R (1983). A global measure of perceived stress. J Health Soc Behav.

[34] Roberti JW, Harrington LN, Storch EA (2006). Further psychometric support for the 10-item version of the perceived stress scale. J Coll Couns.

[35] Boß L, Lehr D, Reis D, et al. Reliability and validity of assessing user satisfaction with web-based health interventions. J Med Internet Res. 2016. 10.2196/jmir.5952. 10.2196/jmir.5952 PMC5023944 27582341

[36] Sidani S, Guruge S, Mirande J (2010). Cultural adaptation and translation of methods: an integrated method. Res Nurs Health.

[37] Skre I, Friborg O, Breivik C (2013). A school intervention for mental health literacy in adolescents: effects of a non-randomized cluster controlled trial. BMC Public Health.

[38] Morgan AJ, Ross A, Reavley N. Systematic review and meta-analysis of mental health first aid training: effects on knowledge, stigma, and helping behavior. PLoS One. 2018. 10.1371/journal.pone.0197102. 10.1371/journal.pone.0197102 PMC5979014 29851974

[39] Wahlbeck K, Aromaa E (2011). Research on stigma related to mental disorders in Finland: a systematic literature review. Psychiatr Fenn.

[40] Hill MR, Goicochea S, Merlo LJ. In their own words: stressors facing medical students in the millennial generation. Med Educ Online. 2018, 2018. 10.1080/10872981.2018.1530558c. 10.1080/10872981.2018.1530558 PMC6179084 30286698

[41] McKerrow I, Carney PA, Caretta-Weyer HC, Furnari M, Juve AM. Trends in medical students’ stress, physical, and emotional health throughout training. Med Educ Online. 2020. 10.1080/10872981.2019.1709278. 10.1080/10872981.2019.1709278 PMC6968533 31902315

[42] Fares J, Tabosh HA, Saadeddin Z, Mouhayyar CE, Aridi H. Stress, burnout and coping strategies in preclinical medical students. N Am J Med Sci. 2016. 10.4103/1947-2714.177299. 10.4103/1947-2714.177299 PMC4791902 27042604

[43] Bruyn S, Wouters E, Ponnet K, Van Hal G. Popping smart pills in medical school: are competition and stress associated with the misuse of prescription stimulants among students? Subst Use Misuse. 2019. 10.1080/10826084.2019.1572190. 10.1080/10826084.2019.1572190 30892122

[44] Salari N, Hosseinian-Far A, Jalali R, et al. Prevalence of stress, anxiety, depression among the general population during the COVID-19 pandemic: a systematic review and meta-analysis. Glob Health. 2020. 10.1186/s12992-020-00589-w. 10.1186/s12992-020-00589-w PMC7338126 32631403

[45] Cranmer GA. One-group pretest–posttest design. In: Allen M, editor. The SAGE encyclopedia of communication research methods. Thoudan Oaks: Sage Publications, Inc.; 2017. 10.4135/9781483381411.n388. Accessed 6 Sept 2021.

