# Correction to: Digital mental health literacy –program for the first-year medical students’ wellbeing: a one group quasi-experimental study

**DOI:** 10.1186/s12909-021-03031-w

**Published:** 2021-12-06

**Authors:** Marjo Kurki, Sonja Gilbert, Kaisa Mishina, Lotta Lempinen, Terhi Luntamo, Susanna Hinkka-Yli- Salomäki, Atte Sinokki, Subina Upadhyaya, Yifeng Wei, Andre Sourander

**Affiliations:** 1 Department of Child Psychiatry, University of Turku, Lemminkaisenkatu 3, FI-20014 Turku, Finland; 2 Finland INVEST Research Flagship, University of Turku, FI-20014 Turku, Finland; 3 ITLA Children’s Foundation, Porkkalankatu 24, 00180 Helsinki, Finland; 4 Department of Nursing Science, University of Turku, Joukahaisenkatu 3-5, FI-20014 Turku, Finland; 5 Turku University Hospital, PO Box 52, 20521 Turku, Finland; 6 Department of Psychiatry, Faculty of Medicine and Dentistry, University of Alberta, 1E1 Walter Mackenzie Health Sciences Centre (WMC), 8440 112 St NW, Edmonton, AB T6G 2B7 Canada

**Correction to: BMC Med Educ (2021) 21:563**

**https://doi.org/10.1186/s12909-021-02990-4**

Following publication of the original article [1], the authors informed us that would like to change the authorship to follow the order first name, last name.

The authorship has been changed above and the original article has been corrected.
