# Supplementary material for: Digital mental health literacy -program for the first-year medical students’ wellbeing: a one group quasi-experimental study
Source: BMC Med Educ. 2021 Nov 6;21:563. doi: 10.1186/s12909-021-02990-4 (PMC8571980; doi:10.1186/s12909-021-02990-4)
Supplement: Supplementary file 1 — Additional file 1: Supplementary Table 1. Background characteristics at baseline of all participants who filled baseline evaluation (n=182) and the participants who filled baseline, post-intervention and follow-up evaluations (n=152). [file 12909_2021_2990_MOESM1_ESM.docx]

**Supplementary Table 1.** Background characteristics at baseline of all participants who filled baseline evaluation (n=182) and the participants who filled baseline, post-intervention and follow-up evaluations (n=152).

| Variable | Count (%)  n=158 | Count (%)  n=182 | *P*-value^a^ | |  |
| --- | --- | --- | --- | --- | --- |
| Year |  |  | |  |  |
| 2018 | 67 (42.4) | 78 (42.9) | | .93 | |
| 2019 | 91 (57.6) | 104 (57.1) | |  |  |
| Gender |  |  | |  |  |
| Female | 117 (74.0) | 133 (73.1) | | .84 | |
| Male | 41 (26.0) | 49 (26.9) | |  |  |
| Age |  |  | |  |  |
| 18-21 years | 108 (68.3) | 128 (70.3) | | .69 | |
| 22-26 years | 50 (31.7) | 54 (29.7) | |  |  |
| Discipline |  |  | |  |  |
| General medicine | 130 (82.3) | 150 (82.4) | | .97 | |
| Dentistry | 28 (17.7) | 32 (17.6) | |  |  |
| Moved from another municipality |  |  | |  |  |
| Yes | 113 (71.5) | 131 (72.0) | | .93 | |
| No | 45 (28.5) | 51 (28.0) | |  |  |
| If yes, when |  |  | |  |  |
| Within previous year | 94/113 (83.2) | 110/131 (84.0) | | .98 | |
| Over a year ago | 19/113 (16.8) | 21/131 (16.0) | |  |  |
| Type of accommodation |  |  | |  |  |
| Alone | 104 (65.8) | 123 (67.7) | | .98 | |
| With a roommate | 11 (7.0) | 12 (6.6) | |  |  |
| With a partner | 33 (20.9) | 35 (19.2) | |  |  |
| With parents | 10 (6.3) | 12 (6.6) | |  |  |
| Help-seeking for mental health problems in previous 3 months |  |  | |  |  |
| No problems | 109 (69.0) | 125 (68.7) | | .99 | |
| Considered or actual help-seeking | 39 (24.7) | 45 (24.7) | |  |  |
| Will not seek help | 10 (6.33) | 12 (6.6) | |  |  |

^a^Chi^2^ -test
